# Longitudinal measurement invariance of the international spinal cord injury quality of life basic data set (SCI-QoL-BDS) during spinal cord injury/disorder inpatient rehabilitation

**DOI:** 10.1007/s11136-021-03027-5

**Published:** 2021-11-03

**Authors:** Simon Kunz, Valerie Carrard, Mayra Galvis Aparicio, Anke Scheel-Sailer, Christine Fekete, Peter Lude, Marcel W. M. Post, Maren Westphal

**Affiliations:** 1 Swiss Paraplegic Research (SPF), Nottwil, Switzerland; 2 Department of Health Sciences and Medicine, University of Lucerne, Lucerne, Switzerland; 3 Present Address: Psychiatric Liaison Service, Lausanne University Hospital (CHUV) and University of Lausanne, Avenue de Beaumont 23, 1011 Lausanne, Switzerland; 4 Swiss Paraplegic Center, Nottwil, Switzerland; 5 Department of Consultation-Liaison Psychiatry and Psychosomatics, University Hospital Zurich, Zurich, Switzerland; 6 School of Applied Psychology, Zurich University of Applied Sciences (ZHAW), Zurich, Switzerland; 7 Department of Rehabilitation Medicine, University of Groningen, Groningen, The Netherlands; 8 Center of Excellence in Rehabilitation Medicine, University Medical Center Utrecht and De Hoogstraat Rehabilitation, Brain Center Rudolf Magnus, Utrecht, The Netherlands; 9 Department of Psychology, Pace University, Pleasantville, USA; 10 Department of Psychiatry, Columbia University, New York, USA

**Keywords:** Spinal cord injuries, Quality of life, Longitudinal measurement invariance, Psychometrics

## Abstract

**Purpose:**

This study aimed at testing the internal consistency and longitudinal measurement invariance of a brief quality of life questionnaire—the spinal cord injury quality of life basic data set (SCI-QoL-BDS)—among individuals with spinal cord injury/disorder undergoing first inpatient rehabilitation.

**Methods:**

Longitudinal data from the Swiss spinal cord injury inception cohort study were used. Participants (*n* = 218) completed the SCI-QoL-BDS at one and three months post injury and at discharge. The SCI-QoL-BDS consists of three items assessing satisfaction with life as a whole, physical health, and psychological health. Internal consistency was examined at each time point and longitudinal measurement invariance was tested using longitudinal confirmatory factor analysis.

**Results:**

Internal consistency coefficients ranged between .82 and .90. The confirmatory factor analysis revealed invariance of the factor structure and of all factor loadings across time. Additionally, all item intercepts except the one of satisfaction with physical health were invariant across time, suggesting partial intercept invariance of the SCI-QoL-BDS. Indeed, a response shift was observed in satisfaction with physical health. This item was evaluated more negatively in the early phase of inpatient rehabilitation, indicating the change of the evolving physical situation after the onset of a spinal cord injury.

**Conclusion:**

The SCI-QoL-BDS is a consistent and valid measure to assess quality of life among individuals undergoing first spinal cord injury/disorder inpatient rehabilitation. However, we recommend using latent variable frameworks instead of mean scores when examining longitudinal changes in the measure to account for potential response shift.

**Supplementary Information:**

The online version contains supplementary material available at 10.1007/s11136-021-03027-5.

## Introduction

Persons sustaining a spinal cord injury or disorder (SCI/D) are at increased risk to experience reduced quality of life (QoL) [1]. SCI/D is a severe impairment caused by physical trauma or disease [2] and the corresponding damage to the neurological tissue typically leads to an immediate, sometimes recovering, sometimes permanent complete or partial loss of body functions, mainly sensor motor and autonomic nerve function below the lesion level [3]. These functional impairments and the risk for developing various secondary health conditions such as chronic pain, fatigue, or muscle spasms [4] as well as resulting restrictions in daily activities and social participation [5] may additionally negatively affect QoL in persons with SCI/D. Thus, besides improving physical functioning and fostering community reintegration, increasing QoL is a key goal of first inpatient rehabilitation following SCI/D [6–8].

Despite general agreement on the clinical relevance of QoL, there is no consensus on the definition of QoL within the SCI/D literature and as a result various measures have been used to assess it [6]. This limits comparability of findings from different studies and creates difficulties to draw any firm conclusions [9]. Therefore, an international group of experts developed the international spinal cord injury quality of life basic data set (SCI-QoL-BDS) [10] with the goal of standardizing the collection and reporting of QoL data in individuals with SCI/D. In addition, the measure aimed to be brief by assessing only a minimal amount of information to facilitate implementation in daily clinical practice. A brief QoL instrument is indeed especially useful in the inpatient rehabilitation phase to efficiently evaluate the evolution of patients’ QoL and the effectiveness of care. The SCI-QoL-BDS is based on a definition of QoL as individual’s subjective evaluation of how things are in their life [8]. This is operationalized with three items asking respondents to indicate to what degree they are satisfied with their life as a whole, their physical health, and their psychological health. Though the authors acknowledged that perceived QoL is a multifaceted construct covering also other domains, these were considered as the most relevant ones in the SCI/D context [10].

Since its development in 2012, the SCI-QoL-BDS has been employed by different research groups around the globe. Accordingly, the original English version was translated into other languages including Dutch [11], Brazilian Portuguese [12], German, French, Italian [13], and Thai [14]. Preliminary evidence from these studies revealed promising psychometric properties of the instrument as indicated by good internal consistency, convergent, and divergent validity among individuals with SCI/D during inpatient rehabilitation [15–17] as well as among community-dwelling individuals [11, 14, 15, 18]. Two longitudinal studies further demonstrated acceptable to good test–retest reliability across a two-week interval [14, 18]. Nevertheless, more examination of the psychometrics properties of the SCI-QoL-BDS during first inpatient rehabilitation are still needed.

Aiming to extend previous research on the psychometric properties of the SCI-QoL-BDS, the objective of the present study was to test the SCI-QoL-BDS’s internal consistency and longitudinal measurement invariance from admission to discharge from SCI/D first inpatient rehabilitation. Testing longitudinal measurement invariance allows to determine whether the instrument assesses the same construct on the same metric at different points in time [19]. This is an important aspect for determining the instrument’s validity, consistency and a fundamental prerequisite to calculate change in the QoL construct and compare its structural relationships with other constructs over time [19, 20]. More specifically, if measurement invariance is not achieved, this indicates that respondents interpret the specific questions and/ or the underlying construct differently at different points in time [21]. As such, changes in scores over time do not necessarily represent quantitative differences in the construct itself. Instead, they may be the result of changes in the meaning of the construct over time (i.e., response shift) or they might be caused by different response styles over time [22, 23]. In sum, examining longitudinal measurement invariance of the SCI-QoL-BDS across first inpatient rehabilitation lays the ground for future research and clinical practice efforts aiming to evaluate the success of rehabilitation practices by measuring changes in the QoL of individuals with SCI/D from admission to discharge.

## Methods

### Participants and procedures

For the purpose of the present study, longitudinal data from the larger inception cohort of the Swiss spinal cord injury cohort study (SwiSCI) [13, 24] were used. The SwiSCI inception cohort is a prospective observational study collecting a wide range of biopsychosocial characteristics of Swiss residents aged 16 years or older, who were newly diagnosed with a traumatic or non-traumatic SCI/D and treated in one of the four Swiss SCI/D rehabilitation centers (Spinal Cord Injury Center of the Balgrist University Hospital, Zurich; Center for Spinal Cord Injury and Severe Head Injury, REHAB Basel; Clinique Romande de Réadaptation, Sion; Swiss Paraplegic Center, Nottwil). SwiSCI excluded individuals whose SCI resulted from congenital conditions (including spina bifida), neurodegenerative disorders (e.g., multiple sclerosis), or happened in the context of palliative care. SwiSCI was approved by the regional ethics committees of all involved Swiss cantons (Ethics Committee northwest/central Switzerland: PB_2016-00183; Ethics Committee Vaud: CCVEM 032/13; Ethics Committee Zurich: 2013-0249) and all participants gave written informed consent.

SwiSCI data collection were conducted by Swiss Paraplegic Research in collaboration with the four Swiss SCI/D rehabilitation centers. During inpatient rehabilitation, measurements were scheduled at one (T1), three (T2), and six months post injury (T3) and at discharge from inpatient rehabilitation (T4). However, because some individuals had a comparatively short first inpatient rehabilitation, not all participants completed each of the measurement time points prior to discharge. Particularly, the six months measurement time point (T3) was missed by many participants due to shorter inpatient rehabilitation duration. Thus, we focused only on the remaining three measurement time points in the present study (i.e., T1, T2, and T4).

**Fig. 1:**
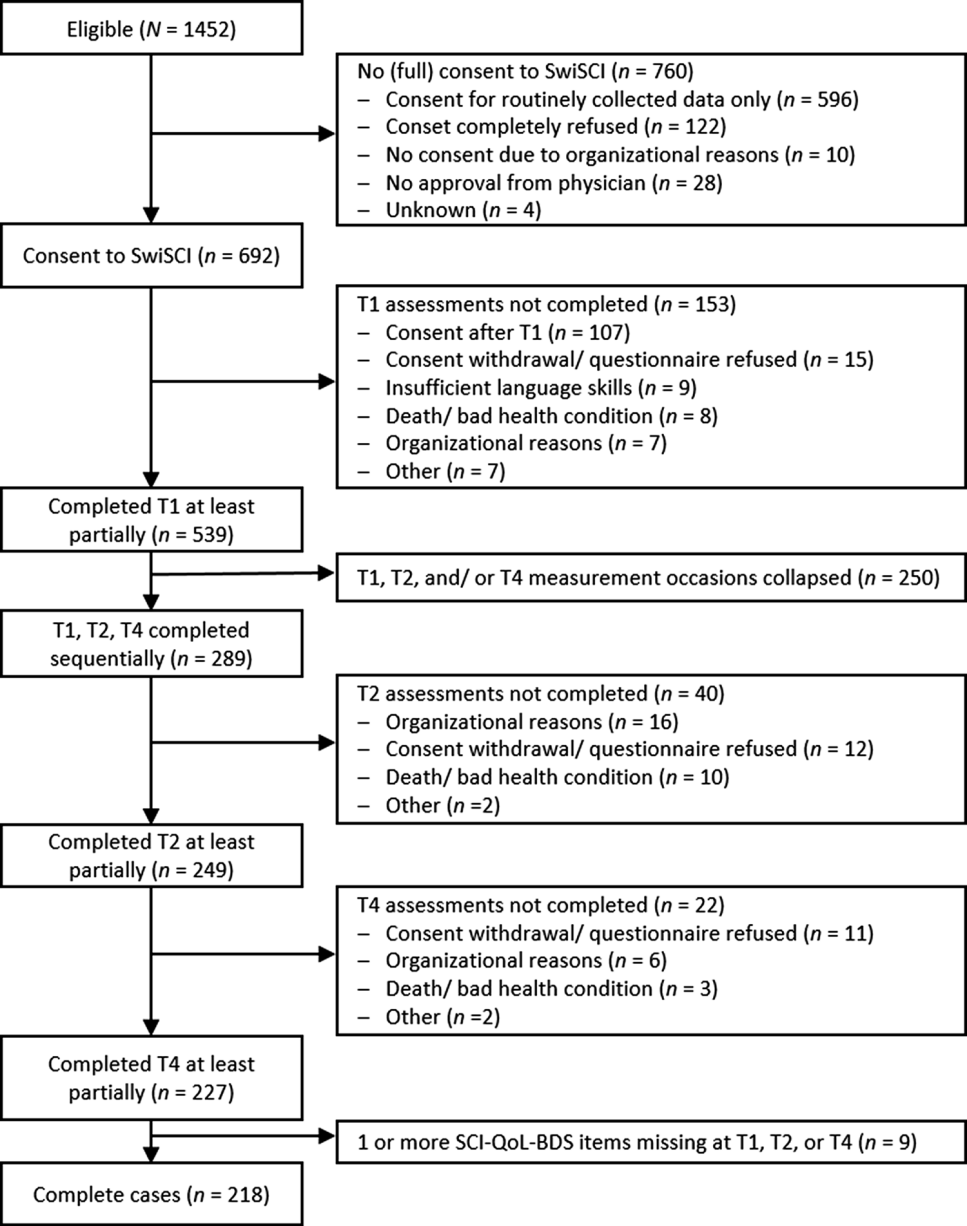
Consort chart depicting participation in the present study

Between May 2013 and January 2021, 1452 individuals were eligible for participation in SwiSCI. Among those, 692 gave their consent for full SwiSCI data collection. We further excluded participants who completely missed T1 (*n* = 153), T2 (*n* = 40), or T4 (*n* = 22) and those for whom one or more of these measurement occasions collapsed (i.e. assessed conjointly at the same time; *n* = 250). Since only 9 (4.0%) of the remaining 227 participants had one or more SCI-QoL-BDS items missing, we decided to run complete case analyses only as little gain can be expected from imputation with such a low amount of missing data [25]. Hence, the final sample size was *n* = 218. Figure 1 specifies the participant flow and reasons for non-participation in more details.

### Measures

The SCI-QoL-BDS consists of three items assessing satisfaction with (a) life as a whole, (b) physical health, and (c) psychological health in the past four weeks [10]. Each item is rated on a numerical scale from 0 (*totally dissatisfied*) to 10 (*totally satisfied*). Item scores can be aggregated into a total SCI-QoL-BDS score by averaging an individuals’ scores across the three items. Higher total scores thereby indicate higher levels in the overall QoL construct. In the multilingual setting of Switzerland, the original English version of the measure as well as its German, French, and Italian cross-cultural translations were used.

Additionally, sociodemographic and injury-related characteristics were retrieved from the patients’ records and used for descriptive purposes. These include sex, time since injury, cause of the injury (traumatic vs non-traumatic), injury level and completeness assessed with the American Spinal Injury Association Impairment Scale (AIS) [26], and physical functioning assessed with the spinal cord independence measure III (SCIM) [27].

### Data analysis

Frequency statistics were used to describe the characteristics of the study sample and of the SCI-QoL-BDS items and total scores. To test the SCI-QoL-BDS’ internal consistency, McDonald’s omega, Cronbach’s alpha and corrected item-total correlations were computed for each time point (T1, T2, and T4). Consistency was considered sufficient if the omega and alpha coefficients were between .70 and .79, good between .80 and .89, and excellent at or above .90. Corrected item-total correlations were considered to be acceptable if they were larger than .30 [28].

Longitudinal measurement invariance of the SCI-QoL-BDS was examined using confirmatory factor analysis. We followed a stepwise procedure, fitting more and more restrictive models to test different aspects of invariance [19, 29]. In the first step, we tested whether the same factor structure of the SCI-QoL-BDS can be established across time (configural invariance).

To do so, a model with minimal identification constraints as described by Widaman et al. [19] was fitted: The three SCI-QoL-BDS items were specified as loading on a common latent QoL factor at each of the three time points with correlations among the residuals of the same item and of the variances of the latent QoL factors across time being allowed [29]. In the second step, we tested whether the factor loadings were equal across time (loading invariance). Finally, in the third step, we tested whether the item intercepts were equal across time (intercept invariance), by sequentially imposing the corresponding longitudinal equality constraints in nested models.

All models were implemented in the Lavaan package in R [30], using the robust maximum likelihood estimator (MLR) to account for the slight non-normal distribution of the variables [31]. Goodness of fit of the configural invariance model was judged by Chi-square (*χ*^2^), comparative fit index (CFI), root mean square error of approximation (RMSEA), and standardized root mean square residual (SRMR). Good model fit is indicated by a nonsignificant *χ*^2^, a CFI above .95, an RMSEA below .06, and an SRMR below .08 [32]. Loading and intercept invariance was evaluated by comparing the corresponding model with the less restrictive one in terms of difference (Δ) in *χ*^2^ and CFI. With regard to Δ*χ*^2^, we used the scaling correction proposed by Satorra and Bentler [33]. A nonsignificant Δ*χ*^2^ and a decrease in CFI of maximally − .005 indicate that the corresponding level of measurement invariance holds [19, 34]. In case full invariance was not supported, partial invariance was tested to locate the source of non-invariance. To do so, we examined the freely estimated unstandardized factor loadings or intercepts and released sequentially the equality constraint for the parameter with the largest difference in the corresponding estimates. Following van de Schoot et al. [21], we repeated this procedure until the model fit was not worse than the one of the less restrictive model, based on the criteria mentioned above. Establishing at least partial intercept invariance allows for a meaningful interpretation of differences in the mean of the latent QoL factors over time as well as their structural relations to other variables [19, 21].

## Results

Descriptive statistics of the study participants are depicted in Table 1. To determine the representativeness of the study sample, participants were compared to non-participants with available data (Supplementary Table 1). No significant differences emerged with regard to physical functioning at T1, level and completeness of the injury. In contrast, participants included in the present study were significantly more likely male, younger, having a traumatic cause of the injury and a longer time to discharge from inpatient rehabilitation. However, the effect sizes in these comparisons were small (Cohen’s *d* between 0.07 and 0.27; Cramer’s *V* between 0.06 and 0.08) indicating only a slight selection bias in the study sample.

**Table 1:** Descriptive statistics of the study sample (*n* = 218)

Characteristic	*n* (%)	*n* (%) missing	*M*	SD
Age at injury		0 (0.0)	51.8	17.1
Sex		0 (0.0)		
Male	162 (74.3)			
Female	56 (25.7)			
Language		0 (0.0)		
German	177 (81.2)			
French	37 (17.0)			
Italian	1 (0.5)			
Other	3 (1.4)			
Time from injury to T1 (days)		10 (4.6)	36.4	6.7
Time from injury to T2 (days)		5 (2.3)	83.1	7.8
Time from injury to T4 (days)		8 (3.7)	174.7	58.6
Cause of SCI/D		0 (0.0)		
Traumatic	142 (65.1)			
Non-traumatic	76 (34.9)			
Level of injury at T1		8 (3.7)		
Paraplegia	135 (61.9)			
Tetraplegia	72 (33.0)			
Unable to determine	3 (1.4)			
Completeness of injury (AIS) at T1		9 (4.1)		
A	48 (22.0)			
B	28 (12.8)			
C	29 (13.3)			
D	103 (47.3)			
E	0 (0.0)			
Unable to determine	1 (0.5)			
SCIM at T1		3 (1.4)	36.7	19.3
SCIM at T4		5 (2.3)	71.7	20.5

*AIS* American Spinal Injury Association Impairment Scale: Grade A = Complete lack of motor and sensory function below the level of injury (including the anal area), Grade B = Some sensation below the level of the injury (including anal sensation), Grade C = Some muscle movement is spared below the level of injury, but 50 percent of the muscles below the level of injury cannot move against gravity, Grade D = Most (more than 50 percent) of the muscles that are spared below the level of injury are strong enough to move against gravity, Grade E = All neurologic function has returned; *SCIM* spinal cord independence measure III

Descriptive statistics of the SCI-QoL-BDS item and total scores are displayed in Table 2. The distributions of the item scores and of the total score were slightly non-normal (Skewness between -0.6 and 0.2; Kurtosis between 2.2 and 2.7) at each of the three measurement time points. Overall, item and total scores tended to increase between inpatient rehabilitation admission and discharge. T-test comparing the SCI-QoL-BDS item and total scores between German-speaking participants and participants speaking other languages showed no statistical difference at any of the three time point.

**Table 2:** Descriptive statistics and internal consistency of the SCI-QoL-BDS items and total score (*n* = 218)

Item	Corrected item-total *r*	McDonald’s omega	Cronbach’s alpha	*M* [95% CI]	SD	Skewness	Kurtosis	Range
Satisfaction with life								
T1	.74	–	–	5.2 [4.8; 5.6]	2.7	0.1	2.2	0–10
T2	.82	–	–	5.6 [5.3; 5.9]	2.5	− 0.2	2.3	0–10
T4	.83	–	–	6.5 [6.2; 6.8]	2.5	− 0.6	3.0	0–10
Satisfaction with physical health								
T1	.68	–	–	4.3 [3.9; 4.6]	2.6	0.2	2.4	0–10
T2	.73	–	–	5.3 [5.0; 5.7]	2.6	− 0.2	2.2	0–10
T4	.82	–	–	5.9 [5.6; 6.2]	2.4	− 0.5	2.7	0–10
Satisfaction with psychological health								
T1	.63	–	–	6.3 [5.9; 6.6]	2.5	− 0.5	2.5	0–10
T2	.71	–	–	6.4 [6.1; 6.7]	2.6	− 0.6	2.4	0–10
T4	.72	–	–	6.9 [6.6; 7.2]	2.4	− 0.6	2.7	0–10
SCI-QOL-BDS total score								
T1	–	.83	.82	5.2 [4.9; 5.5]	2.2	− 0.3	2.4	0–10
T2	–	.88	.87	5.8 [5.5; 6.1]	2.3	− 0.3	2.5	0–10
T4	–	.90	.89	6.4 [6.2; 6.7]	2.1	− 0.5	2.8	0–10

*SCI-QoL-BDS* international spinal cord injury quality of life basic data set; *95% CI* 95% confidence interval

### Internal consistency

Both McDonald’s Omega (between .83 and .90) and Cronbach’s alpha (between .82 and .89) of the SCI-QoL-BDS total score were good at each of the three measurement time points. Item-total correlations were at least *r* = .63 and therefore in the acceptable range (Table 2). Pearson correlations among the SCI-QoL-BDS items are depicted in Table 3. They are exclusively in the moderate to large range [35]. In particular the correlations among the three SCI-QoL-BDS items measured at a specific time point were large.

**Table 3:** Correlations among the SCI-QoL-BDS items (*n* = 218)

Item	1	2	3	4	5	6	7	8
1. Satisfaction with life at T1	–							
2. Satisfaction with physical health at T1	.68	–						
3. Satisfaction with psychological health at T1	.61	.53	–					
4. Satisfaction with life at T2	.48	.49	.50	–				
5. Satisfaction with physical health at T2	.45	.58	.46	.76	–			
6. Satisfaction with psychological health at T2	.38	.34	.51	.73	.61	–		
7. Satisfaction with life at T4	.49	.50	.47	.58	.54	.44	–	
8. Satisfaction with physical health at T4	.42	.45	.41	.54	.55	.45	.82	–
9. Satisfaction with psychological health at T4	.37	.30	.49	.58	.45	.58	.70	.69

All correlations are significant at* p* < .001

### Longitudinal measurement invariance

The fit of the nested models testing different aspects of longitudinal measurement invariance of the SCI-QoL-BDS are shown in Table 4. Except for the significant *χ*^2^, the fit of the configural invariance model tested in the first step was good. All items loaded significantly (*p*’s < .001) on the latent QoL factor at each of the three measurement time points. The corresponding standardized factor loadings were large [35] and ranged between .72 (satisfaction with psychological health at T1) and .95 (satisfaction with life at T2). Taken together, these results indicate that there is configural invariance across inpatient rehabilitation.

**Table 4:** Longitudinal measurement invariance of the SCI-QoL-BDS (*n* = 218)

									Scaled Δ*χ*^2^ difference test				
Model		*χ*^2^	df	*p*	CFI	RMSEA [90% CI]	SRMR	Comp M	Δ*χ*^2^	Δdf	*p*	ΔCFI	Decision
1	Configural invariance	25.31	15	.046	.989	.056 [.014; .091]	.027	–	–	–	–	–	Accept
2	Loading invariance	27.71	19	.089	.991	.046 [.000; .079]	.029	2 vs 1	1.79	4	.774	.002	Accept
3	Intercept invariance	57.52	23	< .001	.964	.083 [.057; .109]	.047	3 vs 2	31.37	4	< .001	− .027	Reject
4	Partial intercept invariance^a^	34.39	22	.045	.987	.051 [.012; .081]	.034	4 vs 2	6.77	3	.080	− .004	Accept

*CFI* comparative fit index; *RMSEA* root mean square error of approximation; *CI* confidence interval; *SRMR* standardized root mean square residual; *Comp M* compared models

^a^Partial intercept invariance model with intercept of T1 satisfaction with physical health freely estimated

After having established configural invariance, we tested the loading invariance model. In this second step, adding the equality constraints on the factor loadings over time did not significantly worsen model fit, as indicated by a scaled Δ*χ*^2^(4) = 1.79, *p* = .774 and ΔCFI = .002. This suggests full loading invariance of the measure over time. We then proceeded with testing the intercept invariance model. As can be seen in Table 4, the intercept invariance model showed a significantly worse fit than the loading invariance model with a scaled Δ*χ*^2^(4) = 31.37, *p* < .001 and ΔCFI = − .027. This indicates that full intercept invariance was not achieve. To identify the source of misfit, we examined the freely estimated intercepts of all items more closely. The largest discrepancy in the unstandardized intercepts emerged for the satisfaction with physical health item at T1 (4.26), which was substantially lower than the ones at later occasions (T2: 5.31; T4: 5.93). Hence, we ran a partial intercept invariance model, releasing the equality constraint on the T1 intercept of the satisfaction with physical health item. This model achieved a fit which was not significantly worse than the one of the loading invariance model, as indicated by a scaled Δ*χ*^2^(3) = 6.77, *p* = .080 and ΔCFI = − .004. Hence, the variability in the measurement of the latent QoL construct can be attributed to the instability of the intercept of the satisfaction with physical health item (see Supplementary Table 2 for the parameter estimates of the partial intercept invariance model).

## Discussion

The present study was the first to examine the internal consistency and different aspects of longitudinal measurement invariance of the SCI-QoL-BDS during first SCI/D inpatient rehabilitation. Supporting results from previous research [11, 14–18], we found good internal consistency of the measure administered at one and three months after injury and at discharge from first inpatient rehabilitation. Using longitudinal factor analyses, we additionally demonstrated that the factorial structure and the factor loadings of the measure were invariant during first inpatient rehabilitation. This suggests that the three items of the SCI-QoL-BDS equally represent a latent QoL construct and that the meaning of this latent QoL factor seems to be stable across time [36, 37]. However, we also found some reasons for caution when using the instrument since only two out of the three items additionally proved to have invariant intercepts over time.

The item which was non-invariant at the intercept level is the respondents’ satisfaction with physical health. For this item, the intercept at one month post injury had to be freely estimated because it was substantially lower than at the two later measurement occasions. This means that the position on the latent QoL construct does not equally transfer to the observed level on this item over time. In other words, there is a systematic tendency for individuals with SCI/D to indicate lower satisfaction with physical health at one month post injury as compared to later measurement occasions and this tendency is not attributable to the concurrent position on the latent QoL variable [36, 37]. This might have resulted from a change in (most of) the respondents internal standards of measurement, so-called recalibration response shift [23, 38]. Individuals might have “recalibrated” their interpretation of the response options for the satisfaction with physical health item over the course of SCI/D inpatient rehabilitation. For example, at the beginning of inpatient rehabilitation, being *totally satisfied* with physical health might have required for individuals to have full physical functioning including the ability to walk and the physical health status before SCI/D onset might be used as reference framework to appraise satisfaction. At later stages of inpatient rehabilitation, individuals may have gained a better understanding of the primary physical consequences of the SCI/D as well as secondary health conditions and thus establish a new reference framework to evaluate their health. At this stage, positive responses to the item *total satisfaction* with physical health may reflect appreciation of absence of secondary health conditions and gains in physical well-being and functioning achieved during inpatient rehabilitation. Further longitudinal research, particularly qualitative studies examining the temporal differences in how individuals with SCI/D evaluate their satisfaction with physical health are needed to understand this potential response shift across inpatient rehabilitation.

As a side note, reevaluating one’s values and criteria for what constitutes good QoL can be an adaptive response to a life-changing event such as SCI/D. Studies of individuals who experienced cancer, loss, or other potentially traumatic events have documented changes in sense of self [39], appreciation of life [40], and life priorities [41]. This raises the possibility that the partial invariance observed in the present study actually might indicate good validity in the sense of reflecting a dynamic cognitive process rather than a static self-assessment, which would not capture the complexity of the psychological adaptation to potentially traumatic events such as the onset of an SCI/D.

The finding of partial intercept invariance has important methodological implications for future longitudinal studies. The response shift seems to take place in the very early phase of inpatient rehabilitation and intercept invariance can be observed in the two later time points. This indicates that the SCI-QoL-BDS might be used without longitudinal measurement issues in studies focusing on the later stages of inpatient rehabilitation spanning from the third month post SCI/D to rehabilitation discharge. Moreover, establishing partial intercept invariance is sufficient to allow for a meaningful interpretation of differences in the latent QoL factor means and their structural relations to other constructs across time [19, 21, 29]. Nevertheless, caution is needed when calculating and interpreting changes in the observed SCI-QoL-BDS total score (i.e., mean score) and in particular in the satisfaction with physical health during the early phase post SCI/D. Comparing observed means would require full intercept invariance [37]. Consequently, a latent variable framework such as latent change score models [42] may be best suited for examining the longitudinal course of QoL and its relationship with other constructs during the early rehabilitation phase [42].

### Limitations

The present study is subject to several limitations. First, it should be noted that we examined the longitudinal measurement invariance of the SCI-QoL-BDS across the inpatient rehabilitation period of individuals with SCI/D. Therefore, it remains unclear whether similar results would emerge when including also measurement time points in the community setting. Hence, future studies are needed to examine the longitudinal invariance of the SCI-QoL-BDS across the whole life span of individuals with SCI/D.

Second, the post hoc investigation of partial invariance at the intercept level was a data-driven approach. As such, it is subject to capitalization on chance [43]. Thus, the present findings should be replicated in future studies with different samples to increase confidence.

Third, a comparison of the characteristics of participants and non-participants with available data indicated a minor selection bias in the present study’s sample. However, individuals who completely refused data collection could not be compared to participants. Hence, some uncertainty regarding the representativeness of the present study sample remains and findings should therefore be interpreted cautiously.

Fourth, a sample size of *N* = 200 is considered to be sufficient for running structural equation models [44]. Nonetheless, with an *N* = 218 our sample size can be considered as modest. Hence, we might have lacked the power to detect weak violations of measurement invariance. As such, future studies with a larger sample size are required to validate the present findings.

### Conclusion

Brief QoL instruments with good psychometrics properties are dearly needed in inpatient rehabilitation settings for an efficient evaluation of the care provided and the recovery of patients. In general, the present study revealed preliminary evidence that the SCI-QoL-BDS is a consistent and valid measure to assess QoL among individuals with SCI/D in clinical research and practice focusing on the inpatient rehabilitation setting. However, our results revealed that the measure might not be fully invariant at the intercept level indicating a recalibration response shift with satisfaction with physical health being comparatively evaluated more negatively in the early phase of SCI/D inpatient rehabilitation. Consequently, we recommend using latent variable frameworks instead of mean scores when examining longitudinal changes from the early stage of SCI/D inpatient rehabilitation to discharge.

## Supplementary Information

Below is the link to the electronic supplementary material.

Supplementary file1 (PDF 154 kb)

## Data Availability

Owing to our commitment to SwiSCI study participants and their privacy, datasets generated during the current study are not made publicly available but can be provided by the SwiSCI Study Center based on reasonable request (contact@swisci.ch).

## Code availability

The analyses’ syntax is available upon request.
