# Supplementary material for: Longitudinal measurement invariance of the international spinal cord injury quality of life basic data set (SCI-QoL-BDS) during spinal cord injury/disorder inpatient rehabilitation
Source: Qual Life Res. 2021 Nov 3;31(4):1247–56. doi: 10.1007/s11136-021-03027-5 (PMC8960613; doi:10.1007/s11136-021-03027-5)
Supplement: Supplementary file 1 — Supplementary file1 (PDF 154 kb) [file 11136_2021_3027_MOESM1_ESM.pdf]

SUPPLEMENTARY MATERIAL

TITLE OF ARTICLE

Longitudinal measurement invariance of the International Spinal Cord Injury Quality of Life Basic Data Set (SCI-QoL-BDS)

JOURNAL

Quality of Life Research

AUTHORS

Simon Kunz*[1,2], Valerie Carrard*[1,2,3], Mayra Galvis Aparicio[1,2], Anke Scheel-Sailer[2,4], Christine Fekete[1,2], Peter Lude[1,5,6], Marcel W. M. Post[7,8], Maren Westphal[9,10]

*Simon Kunz and Valerie Carrard contributed equally to the study and therefore share first authorship.

Affiliations

1. Swiss Paraplegic Research (SPF), Nottwil, Switzerland.
2. University of Lucerne, Department of Health Sciences and Medicine, Lucerne, Switzerland.
3. Psychiatric Liaison Service, Lausanne University Hospital (CHUV) and University of Lausanne, Lausanne, Switzerland
4. Swiss Paraplegic Center, Nottwil, Switzerland
5. University Hospital Zurich, Department of Consultation-liaison Psychiatry and Psychosomatics, Zurich, Switzerland
6. Zurich University of Applied Sciences (ZHAW), School of Applied Psychology, Zurich, Switzerland
7. University of Groningen, Department of Rehabilitation Medicine, Groningen, the Netherlands
8. University Medical Center Utrecht and De Hoogstraat Rehabilitation, Center of Excellence in Rehabilitation Medicine, Brain Center Rudolf Magnus, Utrecht, the Netherlands
9. Pace University, Department of Psychology, Pleasantville, United States
10. Columbia University, Department of Psychiatry, New York, United States

Corresponding author

Valerie Carrard

Current affiliation: Psychiatric Liaison Service, Lausanne University Hospital (CHUV) and University of Lausanne, Address: Avenue de Beaumont 23, 1011 Lausanne, Switzerland. Phone: +41 79 346 42 59

valerie.carrard@chuv.ch

Supplementary Table 1. Comparison between participants and non-participants

	Participants (n = 218)		Non-participants (n = 1234)					
Variable	*M*	*SD*	*M*	*SD*	*t*	*df*	*p*	*Cohen's d*
Age	51.8	17.1	56.7	18.4	3.61	1286	> .001	.27
Time since SCI to discharge (days)	193.7	119.6	162.8	186.4	2.35	1284	.019	.17
SCIM at T1	36.7	19.3	38.5	28.0	0.88	990	.379	.07
Variable	*n*	%	*n*	%	χ^2	*df*	*p*	*Cramer's V*
Sex (Male)	162	74.3	707	66.1	5.60	1	.018	.07
Traumatic cause of SCI	142	65.1	579	54.2	8.85	1	.003	.08
Level of SCI (paraplegia) at T1	135	64.3	505	58.0	4.31	3	.230	.06
Level of SCI (tetraplegia) at T1	72	34.3	345	39.6	-	-	-	-
Level of SCI (intact) at T1	0	0.00	8	0.9	-	-	-	-
Level of SCI (UTD) at T1	3	1.4	16	1.5	-	-	-	-
AIS A at T1	48	23.0	196	22.8	3.47	6	.748	.06
AIS B at T1	28	13.4	92	10.7	-	-	-	-
AIS C at T1	29	13.9	115	13.4	-	-	-	-
AIS D at T1	103	49.2	442	51.4	-	-	-	-
AIS E at T1	0	0.0	7	0.8	-	-	-	-
AIS UTD at T1	1	0.5	8	0.8	-	-	-	-
AIS unknown at T1	0	0.0	1	0.1	-	-	-	-

Note. Only non-participants with available data could be included in the different comparison. Maybe this could be mentioned in the table note UTD = Unable to determine. SCIM = Spinal Cord Independence Measure III. AIS = American Spinal Injury Association Impairment Scale.

Supplementary Table 2. Parameter estimates of the partial intercept invariance model

	Standardized Coefficient	Standard Error	z-value	p-value
Factor loadings				
Latent QoL factor at T1				
Satisfaction with life at T1	0.84	0.13	17.95	<.001
Satisfaction with physical health at T1	0.80	0.12	18.06	<.001
Satisfaction with psychological health at T1	0.71	0.12	14.69	<.001
Latent QoL factor at T2				
Satisfaction with life at T2	0.95	0.13	17.95	<.001
Satisfaction with physical health at T2	0.81	0.12	18.06	<.001
Satisfaction with psychological health at T2	0.73	0.12	14.69	<.001
Latent QoL factor at T4				
Satisfaction with life at T4	0.95	0.13	17.95	<.001
Satisfaction with physical health at T4	0.855	0.12	18.06	<.001
Satisfaction with psychological health at T4	0.745	0.12	14.69	<.001
Intercepts				
Latent QoL factor at T1	0.00	-	-	-
Satisfaction with life at T1	1.97	0.18	29.40	<.001
Satisfaction with physical health at T1	1.62	0.17	24.33	<.001
Satisfaction with psychological health at T1	2.43	0.16	37.76	<.001
Latent QoL factor at T2	0.15	0.07	2.04	.042
Satisfaction with life at T2	2.71	0.18	29.40	<.001
Satisfaction with physical health at T2	1.88	0.19	26.59	<.001
Satisfaction with psychological health at T2	2.45	0.16	37.76	<.001
Latent QoL factor at T4	0.51	0.08	6.27	<.001
Satisfaction with life at T4	2.33	0.18	29.40	<.001
Satisfaction with physical health at T4	2.13	0.19	26.59	<.001
Satisfaction with psychological health at T4	2.66	0.16	37.76	<.001
